# Factors associated with adolescent pregnancy and public health interventions to address in Nigeria: a scoping review

**DOI:** 10.1186/s12978-023-01629-5

**Published:** 2023-06-24

**Authors:** Majesty Enaworoke Alukagberie, Khalifa Elmusharaf, Nuha Ibrahim, Sébastien Poix

**Affiliations:** 1 School of Medicine, University of Limerick, Limerick, Ireland; 2 Public Health Master Programme, University of Limerick, Limerick, Ireland; 3 University of Birmingham, Dubai, United Arab Emirates

**Keywords:** Adolescent pregnancy, Associated factors, Reproductive health, Interventions, Nigeria

## Abstract

**Background:**

Adolescent pregnancy is a global public health and social problem that affects both developed and developing countries. Reducing adolescent pregnancy is central to achieving sustainable development goals. In 2021 Nigeria’s Adolescent pregnancy was 106 per 1000 and showed an increasing rate. This study, therefore, aims to explore the literature to map the risk factors and interventions against adolescent pregnancy in Nigeria.

**Method:**

A scoping review of studies published between January 2007 and December 2022 using PubMed, Web of Science and Africa Journals Online were searched using the keywords' adolescent pregnancy' AND 'Nigeria'. Studies were screened using the eligibility criteria.

**Results:**

A total of 241 articles, of which 229 were identified through the databases and 12 were identified through hand search. After the full-text review, 28 studies met the inclusion criteria and were included in the final review. In Nigeria, the prevalence of adolescent pregnancy is between 7.5 and 49.5%. Associated factors for adolescent pregnancy in Nigeria are multifactorial, including individual, community, societal, school, family, and peer factors. Policies on adolescent sexual and reproductive health exist in Nigeria. Still, the policies need more sponsorship, implementation, and monitoring, while only some interventions on adolescent pregnancy majorly based on contraceptives and education of health providers are available in Nigeria.

**Conclusion:**

Associated factors for adolescent Pregnancy in Nigeria are multidimensional, with educational attainment and wealth index being the highest associated factor. Intervention strategies aimed at the educational level have been identified as a critical factor in curbing adolescent pregnancy. Thus, policies on sexual, reproductive, and mental health development specifically targeting adolescents to reduce the cycle of societal dependence by empowering this group economically and educationally are justifiably warranted.

## Background

Adolescence is a critical period in which significant physical, social, psychological, and reproductive health changes occur between ages 10 and 19 [44]. This time period marks the onset of sexual and reproductive health transformations, including sexual initiation, marriage, and the first child [73]. In sub-Saharan Africa (SSA), approximately one-fifth of the population in 2015 were adolescents and young adults (ages 15–24). Nigeria, in particular, has the highest adolescent population in SSA, accounting for 50 million adolescents, constituting more than one in four youths making up half of Nigeria's current estimated population of 205 million [23]. Thus, these adolescents constitute a significant proportion of the population that undergoes critical SRH transitions over the next decade.

Adolescent pregnancy refers to pregnancy in females between ages 10 to 19 [73] who become pregnant before completing their somatic development [18, 44]. Adolescent pregnancy is often unplanned and considered the most unfavourable outcome of adolescent sexual activity [4, 18]. Globally, approximately 16 million babies are born to adolescent girls between ages 15–19 [68]. In low- and middle-income countries (LMICs), there are an estimated 21 million pregnancies among females under age 19, with 777,000 (6.48%) annual births [20]. The prevalence of adolescent pregnancy in LMICs is 6.48%, with East and Southeast Asia having 7.1% and 33%, respectively [19].

According to World Atlas [21], African countries lead the world in adolescent pregnancy. Mali, Angola, Mozambique, Guinea, Chad, Malawi, and Cote d'Ivoire had 175, 167, 143, 142, 137, 137 and 135 per 100,000 births per year, respectively [21]. In the sub-Saharan African Region, the pooled prevalence of adolescent pregnancy is 19.3%, with East Africa having the lowest prevalence (21.5%) and Northern Africa having the highest (99.2%) [44]. The spatial distribution of adolescent pregnancy in Nigeria ranges from zero to 66.67%, with the North having the highest prevalence [20], even though most adolescent pregnancy in Nigeria occur in union and are intended (an estimated 44% of girls in Nigeria are married before their 18th birthday, and the country ranked the 11th in the world for child marriage [49].

Adolescent pregnancy is a global public health and social issue that impacts both developed and developing nations [45]. Approximately 3.9 million adolescent girls worldwide have unsafe abortions, a significant cause of maternal death and morbidity [29, 36]. In developing countries like Nigeria, adolescent pregnancy is considered the leading cause of newborn and maternal mortality, increased sexually transmitted disease, induced unsafe abortions etcetera [55, 59, 67]. Inequality, health concerns, inadequate public health spending, a low proportion of women in wage work, and low educational success are only a few of the social problems that Adolescent pregnancy has also been linked to [20].

The high rate of adolescent pregnancy in Nigeria has been attributed to various factors, including early onset of menarche among females, early initiation of sexual activity, early marriage, low socioeconomic status, economic insecurity, ineffective use of contraception, low educational and career aspirations, residence in a single-parent home, poor family relationships, and deterioration of traditional African values [4, 69].

Despite the various intervention strategies used to reduce adolescent pregnancy, such as a supportive school environment, school curricula on sexual health, and peer education initiatives [23, 29, 68], the health outcomes of children born to adolescent mothers are still worse than those born to older mothers [30].

However, no study has systematically reviewed the prevalence, associated factors, policies and interventions of adolescent pregnancy in Nigeria, either in the form of a scoping or a systematic review. It is essential to review these variables by pooling all available evidence to develop effective interventions and policies to address the risks associated with adolescent pregnancy, in line with achieving SDG2, SDG3, SDG6, and SDG7 [21, 31, 39]. Nigeria's empirical data to guide national planning for adolescent and mental health are limited [38, 39], and evidence based on empirical studies is crucial to leverage consensus on investing in adolescent health and development for the success of the post-2015 developmental agenda [67].

Nigeria's population is currently estimated at 205 million, with a 3% annual growth rate, and is poised to become the third most populous country in the world by 2050 [23], increasing the need for better prevention and control of adolescent pregnancy. Therefore, understanding the determinant of adolescent pregnancy in Nigeria is crucial to designing, developing, and implementing effective, country-specific interventions and policies. This study aims to determine, review, and characterise the current literature on the elements contributing to adolescent pregnancies in Nigeria to provide better health outcomes for mothers and babies.

## Materials and methods

This scoping review was undertaken to gather evidence on the associated risk factors for adolescent pregnancy in Nigeria. Arksey and O'Malley's methodological framework was followed in conducting this scoping review [16], and the PRISMA-Scr was used in reporting the review (Appendix 1). The five steps recommended by Arksey, and O'Malley were followed in this scoping review. These steps include defining the research question, identifying relevant literature, study selection, data extraction, and lastly, collating, summarising, and reporting the results [16].

### Defining the research question

Papers on adolescent pregnancy in Nigeria published between January 2007 and December 2022 were searched. The choice to include papers from the past fifteen years reflects the urgent requirement for recent data to support advocacy for the domestication and use of WHO guidelines to reduce adolescent pregnancy and enhance adolescent sexual reproductive and mental health in Nigeria. This review used the population, Concept and Context (PCC) approach when formulating the review question.; the population are teenage girls, the concept of Teenage Pregnancy, and the context in Nigeria.

### Identifying relevant literature

#### Search terms and keywords

A brief preliminary search of PubMed and Web of Science (WoS) was recommended to ensure a thorough and extensive search, and African Journals Online (AJOL) using the keywords' adolescent pregnancy' AND 'Nigeria' was first conducted. Then, the index terms used in describing the articles from our preliminary search were analysed to prepare the comprehensive controlled vocabulary for the main search [7].

The Peer Review of Electronic Search Strategies 2015 guideline checklist was used to assess, evaluate, and revise the controlled vocabulary before conducting the main search. Search terms related to the population (e.g., girls, women, adolescents, teenagers, children, young women, young girls, schoolgirls), concept (adolescent pregnancy, teenage pregnancy, childhood pregnancy, girl child pregnancy or unintended pregnancy), and context (Nigeria) were selected. The search strategy used in PubMed is presented in appendix 2.

The following online bibliographic databases were looked through to ensure all pertinent research, PubMed, Web of Science, African Journals Online (AJOL), JSTOR, HINARI, Scopus, PsycINFO, and Science Direct. To ensure appropriate resources, hand searches were also conducted on Google Scholar, WHO, UNICEF, UNFPA, and the Guttmacher Institute websites. The reference list of the discovered review studies was lastly searched.

### Study selection

#### Eligibility criteria

Only English publications and reports published between January 2007 and December 2022 were included in this study. Papers on pregnancy in 10- to 19-year-old adolescents in Nigeria or multi-country studies including Nigeria were included. If publications did not specifically include age-disaggregated data relevant to adolescents, they were excluded. Publications predominantly focused on women of reproductive age (15–49 years) or youths aged 15–35 years were also excluded. Studies that focused on 20–24-year-olds in Nigeria were only considered if they included information on pregnancies that occurred when the participants were between the ages of 15 and 19. Studies that used qualitative, quantitative and mixed-methods research were all included. Only peer-review papers on adolescent pregnancy in Nigeria were included.

#### Screening process

Based on the pre-specified inclusion and exclusion criteria, the reviewer screened all the titles, abstracts, and full texts identified from the initial search. When the reviewer could not decide on the study's eligibility, the assessment of a second reviewer was considered to determine if the study would be included or excluded. All steps and decisions on the study eligibility were recorded in a logbook. Eligible studies were then finally decided through meetings.

### Data charting

A spreadsheet in Microsoft Excel was used to extract information from the full texts. Information extracted included the author's name, the title of the study, the publication year, the design of the study, the sample size, the prevalence of adolescents' pregnancy, observed factors, and the key messages derived from the results, and interventions to reduce teenage pregnancy if used in the study.

### Collating, summarising, and reporting the results

The reviewers used the socioecological model to report the factors mentioned in the articles. The data were organised into common categories and summarised in a table. The results were then reviewed in connection to the general review question through a narrative summary included with the tabulated results. The reviewers used the socioecological model to map Nigeria's factors affecting teenage pregnancy. This model was adopted by [24].

This model describes factors on different levels individual, interpersonal, organisation, community, and policy. This model is used widely to map better factors considering individual factors, social, cultural, policy and systems-related factors [25, 26, 28].

## Results

### Identification and selection of the article

**Fig. 1:**
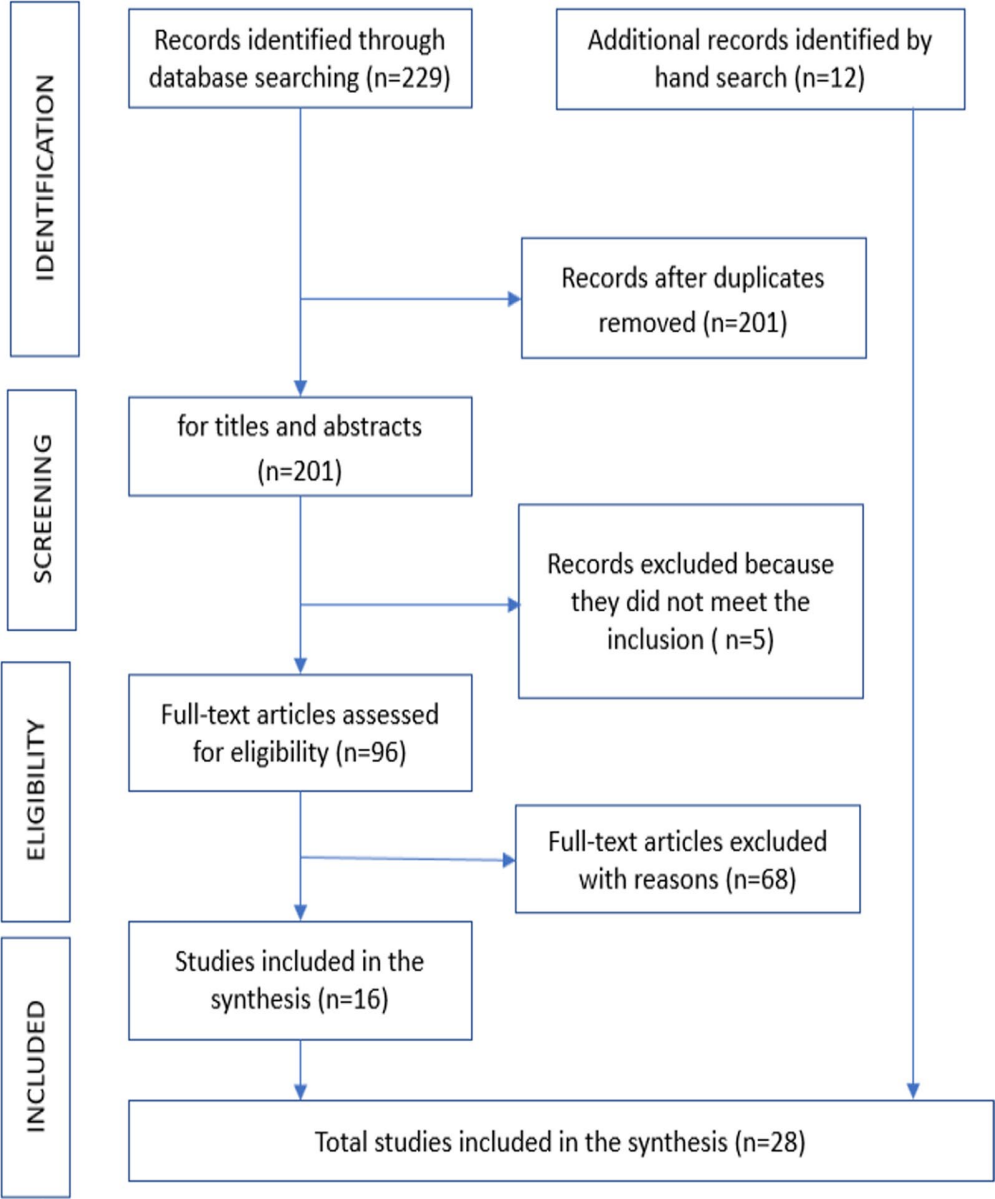
Flow chart describing the selected articles for this scoping review

The initial comprehensive search yielded 241 articles, of which 229 were identified through the databases, and 12 were identified through hand search. After removing 43 duplicates, a total of 201 publications were screened for eligibility based on their titles and abstracts. After reviewing their titles and abstracts, a full-text review of 96 papers was conducted. We removed 68 after doing a thorough text analysis since they did not fit our inclusion criteria for various reasons. Using review article references as a guide, we could not locate any additional articles to include. In the end, 28 publications were included in our scoping review after meeting all inclusion criteria (Fig. 1).

### Characteristics of the studies in the review

Table 1 presents the characteristics of the included articles. A total of 28 studies were included in the study. Twenty-two quantitative studies: 9 descriptive cross-sectional studies out of the 28 included studies [19, 32, 33, 52, 60, 72], 13 comparative cross-sectional studies [1, 6, 18, 34, 45, 57, 58], [8, 10, 21, 36, 62, 63], five qualitative studies :1 focus group discussions [13] and four key informant interviews [11, 35, 48, 64] and one retrospective study [42]. In addition, out of the 28 included studies, 10 were studies that used only the Demographic Health Survey in "Nigeria" [6, 8, 10, 11, 18, 21, 36, 45, 62].

**Table 1:** Characteristics of the selected studies on teenage pregnancy (N=28)

ID no.	Author/year	Study design	Titles	Region in Nigeria	Participants in the study / age	Prevalence of teenage pregnancy	Factors affecting the teenage pregnancy	Interventions
#1	[55]	Descriptive Cross-Sectional.	Contributing variables to teenage pregnancy among female adolescents in Nigeria.	Akoko districts of Ondo State, Southwest	720 pregnant teenagers aged 13-20 years	N/A	Lack of Sexuality Education/Peer Pressure/ Parental Guidance/Media Influence	NA
#2	(O. Alenkhe and J. Akaba 2013)	Mix: Methods: Descriptive Cross-Sectional Survey / Unspecified Qualitative Method.	Teenage pregnancy in Benin city: causes and consequences for future national leaders.	Benin City of Edo state, south south	300 pregnant teenagers <18 years.	12%	Early marriage/ Socioeconomic Status of parents/Peer Influence/Poor Parenting/Unprotected Sex/Religious Affiliation	NA
#3	[1]	Comparative Cross-Sectional Survey.	Unintended pregnancy and childbearing among out-of-school unmarried young women living in Metropolitan city slums, South-West Nigeria.	South-West Nigeria	372 females 10-24 years	N/A	Carelessness and Non-use of contraceptives/ Parenting Failure/ Premarital Sex/ Covetousness/Peer pressure	NA
#4	[20]	Comparative Cross-Sectional Survey.	Spatial distribution and factors associated with adolescent pregnancy in Nigeria: A multi-analysis.	National Study	8448 of females aged 15–19.	N/A	Adolescent Age/ Age of sexual debut/ Education level/Marital status/Working Status /Ethnicity /Religion/ Media Exposure/ Place of residence (Urban/Rural)/ Wealth Index of parents/Sex of the head of the household/ Religion/Community Literacy Level/ Community Socioeconomic Status	NA
#5	[62]	Descriptive Cross-Sectional.	Teenage pregnancy prevalence, pattern and predisposing factors in a tertiary hospital, Southern Nigeria.	South Nigeria	198 Teenage girls 14-19 years.	1.5%.	Older partner/ Low level of education/ Early menarche/ Poor socioeconomic Background / Early Marriage/ Unemployment/ Contraceptive usage	NA
#6	[72]	Descriptive Cross-Sectional.	Teenage pregnancy and its influence on secondary school education in Nigeria.	Obio-Akpor L.G.A, Rivers State,South south	802 Teenage mothers between the ages of 11-18	77%	Education/Peer pressure / Media effect/ Rebellion/ Poverty/ Stability of family/ Alcohol and Drug use	NA
#7	[35]	Secondary Data Analysis of the DHS Data Sets	Trend analysis of teenage pregnancy in Nigeria (1961-2013): How effective is the contraceptive use campaign	National Study	70,811 women <20 years	49.50%	Geographical zone of residence(rural/urban). / Religion/ Education attainment/marital status/ Wealth index/Age at the first marriage/Ethnicity	NA
#8	[8]	Secondary Data Analysis of the DHS Data Sets	Influence of Socio-economic factors on prevalence of teenage pregnancy in Nigeria.	National Study	8,448 Teenage girls < 20 years	19%.	Age. / Place of Residence (rural/Urban)/Religion/ Wealth index of parents/ Educational Attainment/ Marital status/ Employment status/ Visited health facility/ Ask about family planning at health facility	NA
#9	[51]	Comparative Cross-Sectional Survey.	Contemporary factors of teenage pregnancy in rural communities of Abia state, Nigeria.	Abia State, Southeast	400 Adolescents girls aged 11-19 years	49%	Age. / Education/ Marital status/ Employment/Awareness of protective measures against pregnancy/ Parental influence/ Lifestyle and social factors	NA
#10	[46]	Mix: Methods: Descriptive Cross-Sectional Survey / key Informants Interviews.	Causative factors for sexual and reproductive health status of pregnant adolescent girls in urban communities in Lagos.	Lagos State, Southwest	46 Adolescents girl aged 16-19 years old.	5.60%	Peer Pressure. / Poor knowledge of contraceptives/ Curiosity	NA
#11	[6]	Secondary Data Analysis of the DHS Data Sets	Factors associated with teenage pregnancy and fertility in Nigeria.	National Study	6,591 Adolescent girls (<19 years).	7.5% (Currently Pregnant) / 24.1% (Pregnant in the 5 years preceding the survey).	Age/ Educational attainment/ Marital Status. /Religion/ Wealth Index/ of parent Place of residence/ Geographical Area	NA
#12	[31]	Descriptive Cross-Sectional Survey.	Factors predisposing to teenage pregnancy among female adolescents in Isoko south local government area, Delta state, Nigeria.	Delta State, South south	260 Female adolescents <19 years.	N/A.	Knowledge About Teenage Pregnancy. /Peer Influence/ Media Influence/ family support	NA
#13	[43]	Comparative Cross-Sectional Survey.	Socio-demographic risk factors for unintended pregnancy among unmarried adolescent Nigerian girls.	National Study	6,591 adolescents’ girls (15-19 years).	7.50%	Age of Household Head/ Age of the teenage and Educational Attainment / Sex of the household head/ Place of Residence	NA
#14	[58]	Comparative Cross-Sectional Survey.	Teenage pregnancy at a tertiary health institution in South-western Nigeria: Socio demographic correlates and obstetric outcome.	Southwest Nigeria	190 adolescents’ girls < 20 years	1.10%	Education attainment. / Marital Status/ Employment status/ Age	NA
#15	[32]	Descriptive Cross-Sectional.	Factors and conditions that influence teenage pregnancy among in-school adolescents in Umuahia North Local government area of Abia state, Nigeria.	Abia State, Southeast	416 Adolescents girls aged 13-19.	45%.	Financial Gains/ Peer pressure/ Financial difficulties/ Lack of effective sex education/ rape/ Curiosity	NA
#16	[33]	Comparative Cross-Sectional Survey.	Determinants and outcome of teenage pregnancy in a rural community in Jos, Plateau State, Nigeria.	Plateau State, North central	192 Adolescents aged 13-19 years old.	25.5%	Age. / Educational Attainment/Religion/ Marital Status/ Staying with parents/ Still in school/ Family Setting	NA
#17	[9]	Comparative Cross-Sectional Survey.	Regional trends and socioeconomic predictors of adolescent pregnancy in Nigeria: A nationwide study.	National study	22,761 Women aged 15-19.	N/A	Types of residence. / State of Residence/ wealth Index of parent/Age of Respondents/ Educational Level/ Sex of head of the household/ Influence of the media(access to media)	NA
#18	[68]	Descriptive Cross-Sectional Survey.	Intergenerational life courses of teenage pregnancy in Ogbomosho South-Western Nigeria.	Oyo state, Southwest Nigeria	300 Teenagers, both gender	43.50%	Sex of respondents. / Sex of the teenagers/ Age/ religion/ Educational attainment/ marital Status/ occupation/ Disobedience to parent / Low knowledge of sex education and protective methods/ Sexual Harassment/ Lack of parental care/ Financial problem/ Media influence/ No fear of God/Peer influence/ Hawking and laziness/ ignorance of puberty stage (behavioural factors)	Availability of free education Youth forum on sex education Abstinence from sex Religious education Limited family size/number of children Good Parental counselling / proper monitoring and talk
#19	[47]	Descriptive Cross-Sectional Survey.	Causes and effects of teenage pregnancy among female secondary students in Abua/Odual Local Government Area of Rivers State.	Rivers State, South south	1,035 Adolescents girls aged 13-19 years old.	N/A	Lack of information. / Peer pressure/ Pressure from parent/ family of young girls to get married/ Non-use of contraceptive/ Financial and economic factors	Teenage girls’ education Family Love and support for teenage girls Teenage marriage Sex Education in secondary schools Security and avoidance of bad friends
#20	[17]	Comparative Cross-Sectional Survey.	Factors associate with teenage pregnancy and childbearing in Nigeria.		7,819 Adolescents girl aged 13-19.	N/A.	Age. / Education/ Age of the first sex/ Place of Residence/ Region/Religion/ Wealth status/ Marital Status/ type of marriage and family type	NA
#21	[10]	Qualitative approach	Teenage pregnancy in Nigeria: professional nurses and educators ‘perspective [version 1; peer review:2 approved]		80 nurses and teachers	N/A	Ignorance and lack of awareness on sexual education/ Covetousness/ Loneliness/ Low self-esteem / Religious belief /early marriage/ Societal and media pressure/Peer pressure/ Illiteracy/ Rape and incest / Social network / Lack of love or parental guidance/ living with grandparents or relatives/Negative parental Influence/ Polygamy/ Poverty/ Street hawking	Sex education campaigns Sex education in School Sex education in church Abstinence from sex by teenagers Preventative health strategies by using condoms Youth programmes social clubs and groups Provide skills training centres Banning of blue/sex films Religious upbringing Education Improve parents’ child communication Self-discipline Regulations on social media
#22	[53]	Cross-Sectional Survey.	Impact of Timing of sex education on teenage pregnancy in Nigeria: Cross- sectional Survey of secondary school students.	Anambra state	1,234 students Both genders aged 14–17 years and 46 teachers	30%	Financial need/ Poor parental support/ Marital promise/ Peer pressure/ Ignorance/ rape/ sexual abuse/ lack of religious commitment/ Family instability/ Poor use of contraceptives	Early sexual education
#23	[34]	Qualitative approach	Causes, enablers, and perceived solution to teenage pregnancy: a qualitative study in a south –western state in Nigeria.	Ekiti state Southwest Nigeria	25 students (13-19 years) and 8 teachers	23%	Poverty/ Peer pressure/ Child abuse/ Poor parental control.	Abstinence from sexual activities Sex education Governmental awareness programmes Distribution of condoms in school Cordial relationships between parents’ guardians and children
#24	[42]	Retrospective study	Socio-demographic determinants of teenage pregnancy in the Niger Delta of Nigeria.	Bayelsa state, south south	83 teenage girls 14-19 years	6.2%	Age/Educational status/Marital status/Low social class	NA
#25	[69]	Descriptive Cross-Sectional Survey.	Community structure and timing of sexual activity among adolescent girls in Nigeria.	National study	8402 adolescents’ girls <19 years	N/A	Wealth index of parent/Poverty/Gender inequality/community illiteracy level/Education attainment/Community affluence /Community level of women employment/Level of women education.	NA
#26	[59])	Qualitative (in depth interview)	“It’s like being involved in a car crash”: teen pregnancy narratives of adolescents and young adults in Jos, Nigeria	Plateau state North central	17 adolescents and young women 16-24 years	8.2%	Lack of sexual and reproductive health education	NA
#27	[63]	Descriptive Cross-Sectional Survey.	Unintended pregnancy and termination of studies among students in Anambra state, Nigeria: are secondary schools playing their part?	Anambra state Southeast	1234 students (14-17 years) and 46 teachers Both gender	N/A	Lack of sex education	Counselling, Abstinence from premarital sex, good company and joining of religious groups, Sex education, Proper parental upbringing, Adequate financial provision of female students, and Involvement of PTA advocacy group.
#28	[57]	Cross-Sectional Survey.	Socioeconomic inequalities in teenage pregnancy in Nigeria: evidence from Demographic Health Survey	National study	8423 of women 15-19 years	N/A	Teenage education level, Marital status, Religion, Occupation, Place of residence, Geopolitical zone, Wealth index quintiles, and exposure to information and communication technology (ICT) (frequency of watching television and use of internet)	NA

### Geographical region in Nigeria

In terms of the geographical location of the studies, six studies were conducted in South-west Nigeria [1, 35, 48, 60, 63, 72], while five were conducted in South-south [14, 32, 52].On the other hand, few studies (4) were conducted in the Southeast [19, 33, 57, 58], and finally, only two studies were conducted in North Central [33, 62].

### Prevalence of adolescent pregnancy

Fourteen out of the twenty-eight studies included in the analysis reported adolescent pregnancy prevalence. In Nigeria, the prevalence of adolescent pregnancy, as recorded by researchers, was 7.5%, 19%, 23%, 7.5–24%, 30%, and 49.50% [6, 8, 35, 36, 45, 58]. The regional prevalence, as reported by researchers in South-south, was 1.5%, 12% and 77% [14, 52], South-west 1.1%, 5.60% and 43.50% [48, 63, 72], Southeast 45% and 49% [32, 55] and North 14.30% and 8.2% [33, 62] (Table 1).

### The design of the selected studies

Twenty-eight studies were included in the study. Twenty-two studies were quantitative (9 descriptive cross-sectional and 13 comparative cross-sectional studies), while five were qualitative studies and one retrospective study (Table 1).

### Factors predicting adolescent pregnancy in Nigeria

The factors were categorised using the social-ecological model [24] into the individual level, interpersonal level factor, organisational level and community-level factors were identified as correlate/putative risks for adolescent pregnancy in Nigeria (Table 2).

**Table 2:** Social-ecological model of the associated factors of adolescent pregnancy in the selected studies

Associated factors	Number	%	Articles ID
*Individual level*			
Occupation	5/24	21	#8, #16, #17, #18, #21
Rape	6/24	25	#15, #18, #21, #22, #23
Employment status	4/24	13	#5, #9, #14, #25
Education attainment	18/24	75	#4, #5, #6, #7, #8, #9, #11, #13, #14, #16, #17, #18, #20, #21, #24, #25, #26, #28
Financial gains	5/24	21	#15, #18, #21, #22, #25
Rebellion/disobedience	3/24	13	#13, #18, #21
Age	12/24	50	#4, #5, #7, #8, #11, #13, #14, #16, #17, #18, #20, #24
Alcohol and drug use	1/24	4	#6
Contraceptive use	6/24	25	#2, #3, #5, #10, #19, #22
Ignorance of the pubertal stage	9/24	38	#1, #3, #10, #12, #15, #18, #19, #21, #22
Curiosity	1/24	4	#15
Early menarche	1/24	4	#5
*Interpersonal level*			
Marital status	14/24	58	#2, #4, #5, #7, #8, #11, #13, #14, #16, #18, #20, #22, #24, #28
Wealth index of parents	14/24	58	#2, #4, #5, #6, #7, #8, #11, #17, #18, #19, #20, #21, #23, #25
Peer pressure	9/24	38	#1, #2, #3, #6, #10, #15, #19, #21, #23
Parental support	7/24	29	#1, #2, #12, #18, #21, #22, #23
Sex of household head	2/24	8	#13, #17
Family background	7/24	29	#9, #16, #17, #19, #20, #21, #22
Media influence	8/24	33	#1, #4, #6, #12, #17, #18, #21, #28
*Organisational level*			
Religion	11/24	49	#2, #4, #7, #8, #11, #16, #18, #20, #21 #22, #28
*Community level*			
Community literacy level	2/24	8	#4, #25
Place of residence	7/24	29	#4, #7, #11, #13, #17, #20, #28
Geographical region	5/24	21	#4, #7, #11, #20, #28
Ethnicity	3/24	13	#4, #7, #13

### Interventions of adolescents pregnancy in the selected studies in Nigeria

Six out of the twenty-eight studies included in the analysis reported interventions of adolescent pregnancy in Nigeria [11, 19, 35, 58, 72]. The interventions are grouped into individual level which include abstinence from sexual activities, self-discipline, preventive health strategies and avoidance of bad friends [11, 35, 72] governmental level which include government awareness , regulation of social media, banning of blue film etc [11, 19, 35, 72] interpersonal level which includes adequate financial support , Proper parental upbringings and guidance and cordial relationships between parents and children etc [11, 19, 35, 72] organisational level which include distribution of condoms in schools, joining religious groups and religious upbringing [11, 19, 35] ) and educational levels reported as sex education [11, 19, 35, 58, 72] (Table 3).

**Table 3:** The interventions to address the AP in Nigeria

Interventions	Numbers	%	Articles ID
*Individual level*			
Abstinence from sexual activities	4/20	20	#18, #21, #23, #27
Self-discipline	1/20	5	#21
Preventive health strategies by using condoms	1/20	5	#21
Security and avoidance of bad friends	1/20	5	#19
*Governmental level*			
Governmental awareness programmes	2/20	10	#21, #23
PTA advocacy group	1/20	5	#27
Regulation of social media	1/20	5	#23
Banning of blue films/ sex films	1/20	5	#21
Provide skills training centres	1/20	5	#21
Youth programme, social club, and group	2/20	10	#18, #21
*Interpersonal level*			
Cordial relationships between parents, guidance, and children	2/20	10	#21, #23
Adequate financial support	1/20	5	#27
Proper parental upbringings	2/20	10	#18, #27
Limited family size/no of children	1/20	5	#18
Family love and support for teenage girls	1/20	5	#18
Teenage marriage	1/20	5	#18
*Organisational level*			
Distribution of condoms in schools	1/20	5	#23
Joining of religious group	1/20	5	#27
Religious upbringings	1/20	5	#21
*Educational level*			
Sex educational	6/20	30	#18, #19, #21, #22, #23, #27

## Discussion

This scoping review offers a comprehensive evaluation and synthesis of data from 28 research articles that concentrate on associated factors and interventions of adolescent pregnancy.

### Prevalence of adolescent pregnancy in Nigeria

The prevalence of adolescent pregnancy in Nigeria ranged from 7.5% to 49.5%. The prevalence was found to vary by region, with the Northcentral region having the highest prevalence of 14.3% and the South-south region having the lowest prevalence of 5.2%. These findings are consistent with previous studies that have shown regional differences in adolescent pregnancy rates in Nigeria [73]. This prevalence is higher than other SSA, which ranged from 7.2% in Rwanda to 44.3% in Congo [5]. The attitudes and beliefs of individuals and society towards the education of the female child, public health campaigns and the promotion of contraceptive uptake, as well as religious and cultural norms, can significantly impact the prevalence of adolescent pregnancy. For instance, in some parts of Nigeria, the education of the female child is not prioritized, and girls may have limited access to information about sexual and reproductive health. Furthermore, some religious and cultural practices may discourage the use of contraceptives, leading to a higher risk of unintended pregnancy. [5]. To further support this finding, a study by Ilene S Speizer et al. [73] revealed that in northern Nigeria, girls have lower levels of education, are more likely to be married early, and have less access to healthcare and family planning services, compared to their southern counterparts. On the other hand, in the southern region, where there is generally more education and access to healthcare, adolescent pregnancy prevalence is lower.

### Individual factors influence adolescent pregnancy in Nigeria

#### Age at first sex

Early adolescent sexual activity has been identified as a significant factor contributing to the high prevalence of adolescent pregnancy in Nigeria. Studies have shown that a higher incidence of multiple sexual partners, unprotected sex, unwanted and adolescent pregnancy, and unsafe abortions are associated with early sexual debut [8, 33]. Bolarinwa et al. [20] reported that a high level of adolescent pregnancy was noted in adolescents who had a sexual debut between the ages of 15–19 years. This could be due to the fact that young adolescents at that age struggle to meet their fundamental needs such as economic resources, education, and proper guidance [20]. Therefore, delaying sexual debut and promoting safe sexual practices among adolescents through sex education and access to contraceptives can help reduce the incidence of adolescent pregnancy in Nigeria.

#### Married adolescents

Marriage is a significant factor associated with adolescent pregnancy in Nigeria, as married adolescents are more likely to get pregnant than unmarried ones [20]. In the northern region of Nigeria, where social norms are less supportive of female education and contraceptive use, and early marriage is encouraged, parents may coerce their unmarried adolescent daughters into marriage to avoid bringing shame to the family [20]. The probability of adolescent marriage increases due to the poor socioeconomic status of the family and economic recession following the loss of household income, as in cases of bad health, accidents, pandemics (such as COVID-19 lockdowns), and retrenchment [35].

Nigeria is a signatory to the FP2020 summit made by the Economic Community of West African States (ECOWAS) member states to eliminate child marriage while promoting various forms of Adolescent Sexual Reproductive Health and Rights (ASRHR) [48]. However, not all states have included laws banning child marriage in their state laws, leaving young girls vulnerable to early marriage and pregnancy. Efforts should be made to enforce laws banning child marriage and to promote education and contraceptive use among girls.

#### Educational attainment

Educational attainment is a crucial factor related to adolescent pregnancy in Nigeria. Research studies have shown that adolescent pregnancy is higher among primary school holders compared to secondary school holders [17]. Educated adolescents in Nigeria have better access to knowledge and resources, which allows them to delay childbearing and marry later [61].

However, poor education correlates with poor knowledge about reproductive health and practices, which leads to poor sexual and reproductive health outcomes. Factors such as poor sex education, the cost and unavailability of contraceptives, and negative attitudes of healthcare providers towards adolescents seeking contraception contribute to poor contraceptive use by adolescent girls in Nigeria [8, 41]. Additionally, misconceptions around the use of contraceptives, such as the perception that only boys/men should buy condoms [74] and that contraceptives are reserved only for married couples [62], perpetuate low contraceptive use among adolescent girls in Nigeria.

To address these issues, there is a need to integrate comprehensive sex education (CSE) into formative schools' curricula in Nigeria. Efforts have been made to address these issues in Nigeria, with the government allocating $3 million annually for the purchase of reproductive health goods in 2015, and an additional annual increase of $8.35 million from 2016 to 2019 [35]. Several donor agencies, such as UNFPA/UNICEF, are also providing modern contraceptives in health facilities in Nigeria [56]. Additionally, the "A360 9ja Girls" intervention is addressing the low rates of contraceptive use among adolescent girls in ten states in southern Nigeria [60].

#### Peer pressure

Peer pressure has been identified as a significant factor influencing adolescent pregnancy in Nigeria, as evidenced by several studies [1, 13, 14, 52, 60]. Adolescents are often influenced by their peers, who can serve as agents of socialization and shape their attitudes and behaviors towards sexual activity [52]. Peer groups that support early sexual activity may normalize and encourage risky sexual behavior among adolescents.

Moreover, some adolescents believe that engaging in sexual activity is a normal casual behavior, and those who abstain are seen as being abnormal. This misconception is often perpetuated by peer pressure and can lead to an increase in adolescent pregnancy rates. Additionally, peers can also influence their group's views, leading to risky behaviors such as drug and alcohol abuse that can result in unintended pregnancy.

To address this issue, there is a need to educate adolescents on healthy relationships, sexual behaviors, and the risks associated with early sexual activity. Parents and caregivers can play a critical role in discussing sex and sexuality with their children and providing them with accurate information. Additionally, schools can incorporate sex education into their curricula, focusing on healthy relationships, contraception, and responsible sexual behavior. It is also important to create safe spaces for adolescents to discuss their sexual health concerns and to provide them with access to contraception and other reproductive health services.

#### Parental support

It is important to note that parental support can play a protective role in reducing adolescent pregnancy [31]. Poor parenting and parental neglect often result in a lack of communication and guidance on sexual and reproductive health matters. This leaves adolescents vulnerable to misinformation from their peers and other sources, which can lead to risky sexual behaviors and unintended pregnancies [68]. Parents may prioritize economic benefits for the family over caring for and guiding their children, especially girls, which can lead to neglect and increased peer influence [3]. Furthermore, parental counseling and guidance can provide a safe and supportive environment for adolescents to discuss sexual and reproductive health issues and receive accurate information. This increased parental guidance and community awareness have contributed to the reduction in the prevalence of adolescent pregnancy in some areas of Nigeria. [20, 70]. However, more efforts are still needed to ensure that parents are adequately equipped with the necessary knowledge and skills to provide the needed guidance to their adolescent children on sexual and reproductive health matters. Additionally, there is a need to ensure that communities are better informed about adolescent pregnancy and its consequences, as well as the importance of preventing it through appropriate interventions such as comprehensive sex education, availability of contraceptives, and access to healthcare services.

#### Single parenting

Single-parenting, particularly in families headed by a male head, has been identified as a factor associated with adolescent pregnancy in Nigeria [9, 55]. Adolescent pregnancy has been found to be more prevalent in families with male heads, possibly because they may struggle to build trust with and engage adolescent girls in sensitive dialogues about their sexuality and warn them of potential dangers [9]. It highlights the importance of involving male heads of families in adolescent reproductive health and family planning programs to improve their understanding and support of their daughters in these areas.

#### Media exposure

It is important to note that media exposure is a complex issue in Nigeria and can have both positive and negative impacts on adolescent sexual and reproductive health [10, 32, 72]. While media content can perpetuate negative stereotypes and promote risky behavior [40], it can also be a powerful tool for disseminating accurate information and promoting positive social norms [26].

Efforts should be made to ensure that media content is culturally sensitive, age-appropriate, and evidence-based to promote healthy sexual and reproductive behaviors among adolescents in Nigeria. This could include the promotion of comprehensive sex education in media content, and collaboration with media organizations to develop and disseminate accurate and reliable information about sexual and reproductive health.

#### Socioeconomic status

The socioeconomic status of the family plays a crucial role in adolescent pregnancy in Nigeria [10, 11, 21, 35, 52]. Research has shown that families with a low wealth index are more likely to have adolescent girls who become pregnant than those from wealthy families. Poverty influences other factors such as transactional sex, multiple sexual partners, and early marriage, which increase the risk of adolescent pregnancy [32, 43]. This pattern is seen across all geopolitical zones in Nigeria, where girls from impoverished households have unmet social needs and are more likely to become pregnant than those from high socioeconomic backgrounds [9]. Additionally, girls from low-income families may engage in transactional sex as a way to escape financial struggles, further increasing their risk of becoming pregnant [32, 70]. It is essential to address the issue of poverty in Nigeria and provide support to families with low socioeconomic status to prevent adolescent pregnancy.

#### Rural-urban differentials

Rural-urban differentials are a community factor for adolescent pregnancy in Nigeria [9, 35]. A higher proportion (57.4%) of rural adolescents compared to 42.3% of their urban counterparts become pregnant before 20 years [17]. Violent neighborhoods’ making girls perceive an attack, seeking male protection, and living in crowded places (like slums) can lead to adolescent pregnancy [17].

The region of the country significantly influences adolescents’ pregnancy in Nigeria [8, 21, 36]. The geographical region often affects the literacy level of the community. From studies in Nigeria, the Northwest and Northeast appear to have more pregnant adolescents than other regions [8, 20]. This may be attributed to more population of poorly educated/illiterate and socioeconomically disadvantaged adolescent girls in the region [20], as well as the sociocultural/customary and religious practices like early (soon after menarche) marriages practised in Islam which is more prevalent in the northern States of Nigeria [35]. However, South-south and South-west also had more adolescents pregnancy than other regions [8]. Moreover, in this present study, Southeast had a higher prevalence, thus indicating that the problem of adolescent pregnancy is not peculiar to a particular part of Nigeria.

#### Religion

In this review, religion was found to be an organsiational factor for adolescent pregnancy in Nigeria. The sexual behaviours of young people are dependent on the tenets and doctrines of their religious affiliations, making the integration of comprehensive sex education (CSE) in school curricula complex in Nigeria [8]. The integration of a CSE in school curricula in Nigeria is complex as some topics offered in CSE, such as contraception, and sexual orientation potentially contradict the beliefs of some religious groups in the country [8]. Studies have reported that girls practicing Islamic religion were more prone to adolescent pregnancy as many of them are susceptible to early marriage and may be resistant to education on contraceptives [8, 17].Therefore, religious leaders should play a critical role in interventions that are sensitive to religious beliefs and ethnic peculiarities [14].

#### Ethnicity

Ethnicity is a personal risk factor for adolescent pregnancy in Nigeria[51]. Adolescence is culturally defined in Africa, including Nigeria, which has resulted in a negative attitude of parents toward sex education, creating mistrust and preventing girls from seeking or disclosing sensitive information to secure the social or clinical support they need [20]. In Nigeria, preferential support is given to males perpetuating gender inequality and contributing to the lack of education and gender-based violence experienced by female adolescents. Adolescent girls from the Igbo ethnic group are particularly at high risk of pregnancy, as the patriarchal traditions of the Igbo culture view men as superior and more dominant than women, leading to situations where girls in sexual relationships with men are least likely to refuse sexual activity [20]. This situation similarly exists in sexual relationships between an adolescent girl and an older male partner as well as in coerced sex in cases of rape, trafficking, fostering, and war [32, 65]. Additionally, gender-based violence, which is often present in abusive relationship, also predisposes adolescent girls to pregnancy [11]. Despite the existence of organisations, laws, policies, action plans, there is currently no programme addressing gender-based violence in Nigeria [48]. Moreover, customarily, girls are perceived as instruments for procreation, leading parents to push their girls, including adolescents, to become pregnant [51].

### Intervention strategies to halting adolescent pregnancy

This review has revealed some factors associated with adolescent pregnancy in Nigeria and identified some intervention strategies for curbing this issue. These strategies can be grouped into individual, governmental, interpersonal, organisational, and educational levels.

#### Sexual education

Sexual education at home, in schools, churches or mosques is a primary strategy for curbing adolescent pregnancy in Nigeria, as emphasized by several researchers [11, 35, 58, 72]. It is crucial that sexuality education covers important topics such as contraceptives, HIV/AIDS study, family health and reproductive health. The result of this study gives credence to the work of other researchers that good and early sex education is a significant intervention against adolescent pregnancy in Nigeria [15, 55]. This approach has shifted the norm in many countries where girls were expected to leave school to start a family, emphasizing instead gender equity and girls' education, which enables them to delay marriage and childbearing. Consequently, gender-equitable and sexual reproductive health norms have improved, reducing gender-based violence and promoting equitable behaviors and attitudes among community members.

While good sexual education is necessary to prevent adolescent pregnancy, it is also essential to provide constant training of teachers on reproductive health to enable them to impact better knowledge on students [2]. Additionally, parents should receive education to overcome cultural barriers that discourage the provision of sex education to their children. This strategy has helped improved norms related to discussing sexual reproductive health among teachers and health workers, leading to better outcomes for adolescents.

#### Abstinence from sex

This scoping review has found that abstaining from sex is a one of the strategies adopted for reducing adolescent pregnancy in Nigeria, as supported by previous studies [11, 35, 72]. Encouraging adolescents to abstain from sexual activity can help delay the onset of sexual activity, reduce the number of sexual partners, and ultimately decrease the risk of unintended pregnancy. However, it is important to acknowledge that promoting abstinence-only education without providing information about contraception may not be effective, and comprehensive sexuality education should be integrated into interventions to ensure adolescents have access to accurate and reliable information about reproductive health.

#### Good parenting

Good parenting and effective parent-child communication is another important intervention for adolescent pregnancy in Nigeria [11, 35, 72]. This approach involves providing parents with the knowledge and skills to communicate with their children about sex education and reproductive health, as well as establishing open and supportive relationships. Scaling up this strategy has helped to improve the quality and frequency of parent-child discussions on sexual reproductive health topics and has also led to the adoption of more positive norms related to discussing these issues with the community.

#### Eradicating under-aged marriage

Eradicating under-aged marriage is a crucial strategy in reducing adolescent pregnancy rates in Nigeria [47]. This can be achieved through various means, including the provision of skill training centers and enrollment in schools [10, 47]. This approach has been successful in improving gender norms related to early marriage and reducing its prevalence. By delaying marriage, girls have more opportunities to pursue education and establish their careers, leading to better reproductive health outcomes [47]. The eradication of under-aged marriage also aligns with the United Nations Sustainable Development Goals, specifically goal 5: gender equality and empowering women and girls, which emphasizes the elimination of all harmful practices, such as child marriage, that affect the well-being of women and girls.

### Strengths and limitations

Our review has several strengths, including the use of a rigorous methodology to address the research questions. However, it is important to note that only studies published in English were included, which may have limited the scope of our findings. Some studies included in our review had small sample sizes, which could have led to an underestimation of the prevalence of adolescent pregnancy in Nigeria. Additionally, our review did not examine the subcomponents of the interventions to better understand the causal pathways to the impact of the studies that recorded successful interventions. Therefore, the replication or scaling up of these successful interventions should be done with caution. Lastly, our focus on research conducted solely among adolescents in Nigeria may have excluded potentially successful interventions recorded in other settings that could be adapted to Nigeria.

### Study implication for policy and research

To effectively leverage international attention and improve interventions against adolescent pregnancy in Nigeria, it is essential to consider the empirical evidence from this scoping review on the prevalence, associated factors, policies, and interventions for preventing adolescent pregnancy. This evidence can inform the development and investment in Adolescent Sexual and Reproductive Health and Rights (ASRHR) policies and programs in Nigeria. Implementing evidence-based interventions is expected to yield a high return on investment and positively impact the sexual and mental health of adolescents. Policymakers, researchers, and stakeholders should use the findings from this review to inform policy decisions, design effective interventions, and conduct further research to strengthen the evidence base on interventions against adolescent pregnancy in Nigeria.

## Recommendations

The findings of this scoping review have highlighted the need for effective interventions to address the issue of adolescent pregnancy in Nigeria. Based on the evidence presented, a number of recommendations are proposed to improve ASRMH in Nigeria. These recommendations focus on multisectoral approaches to address structural issues, provision of adolescent-friendly sexual and reproductive education, improving parenting skills, and the need for further research to inform evidence-based interventions. By implementing these recommendations, Nigeria can take significant steps towards reducing the prevalence of adolescent pregnancy and improving the overall health and well-being of its young population.

1. *Develop and implement evidence-based interventions*: Policymakers, healthcare professionals, and other stakeholders in Nigeria should develop and implement evidence-based interventions using innovative approaches to prevent adolescent pregnancy. Such interventions should include multisectoral approaches that address structural issues like education, poverty, gender-based violence, and lack of economic opportunity, which underlie poor ASRMH. Innovative approaches, such as mobile health technology or social media, should be explored and evaluated for their potential effectiveness in improving adolescent sexual and reproductive health outcomes.
2. *Increase access to adolescent-friendly sexual and reproductive education*: There is a need to increase access to adolescent-friendly sexual and reproductive education, including contraception, and encourage girls to receive at least secondary level education to potentially serve to delay age at the birth of the first child. This can be achieved through the integration of comprehensive sexuality education (CSE) in the curricula of primary and secondary schools in Nigeria.
3. *Provide massive public education*: Mass public education campaigns can also be used to raise awareness about the negative consequences of adolescent pregnancy and the need for preventive measures. This can be done using mass media, traditional/community, religious and sports gatherings, peer-to-peer education, etc.
4. *Improve parenting skills:* As good parenting skills are critical for adequate infant growth and development, interventions aimed at improving parenting skills should be a key component in developing care models and intervention packages for adolescents in Nigeria. Such interventions can be designed to enhance parent-child communication on reproductive health and other sensitive issues.
5. *Join the Global Programme to End Child Marriage*: Nigeria should consider joining the Global Programme to End Child Marriage (GPECM) as this can help to reduce adolescent pregnancy in the country. The GPECM provides technical assistance and resources to support countries in implementing evidence-based interventions to end child marriage and promote gender equity.
6. *Further research:* Research is needed to better understand the specific contextual factors that contribute to adolescent pregnancy in different regions of Nigeria, as well as the effectiveness of interventions tailored to these contexts. Long-term monitoring and evaluation of adolescent pregnancy prevention programs and interventions are necessary to assess their impact over time and identify areas for improvement. The government, donors, and NGOs should prioritize funding for adolescent sexual and reproductive health research, in order to address the ongoing issue of adolescent pregnancy in Nigeria.

## Conclusion

In conclusion, this scoping review has highlighted the significant prevalence and diverse risk factors associated with adolescent pregnancy in Nigeria. It has also provided insight into intervention strategies at various levels that can help curb this issue. However, the policies on Adolescent Sexual and Reproductive Health (ASRH) in Nigeria still lack the necessary support and implementation, and existing policies often discriminate against unmarried pregnant and under 18-year-old adolescents. It is crucial for Nigeria to take a multisectoral approach in addressing the structural issues that underlie poor ASRMH and invest in evidence-based interventions to improve the sexual and mental health of adolescents. More research is needed in all geopolitical regions of Nigeria to provide a more comprehensive understanding of adolescent pregnancy in the country. If implemented effectively, the recommendations provided in this review can go a long way in curbing adolescent pregnancy, leading to better socioeconomic development and improved health outcomes for adolescents and their children in Nigeria.

## Data Availability

All data generated or analysed during this study are included in this published article.

## Appendix 1

**Table:**

Section	Item	PRISMA-ScR checklist item	Reported on page #
*Title*			
Title	1	Identify the report as a scoping review.	Cover page
*Abstract*			
Structured summary	2	Provide a structured summary that includes (as applicable): background, objectives, eligibility criteria, sources of evidence, charting methods, results, and conclusions that relate to the review questions and objectives.	3
*Introduction*			
Rationale	3	Describe the rationale for the review in the context of what is already known. Explain why the review questions/objectives lend themselves to a scoping review approach.	4–6
Objectives	4	Provide an explicit statement of the questions and objectives being addressed with reference to their key elements (e.g., population or participants, concepts, and context) or other relevant key elements used to conceptualize the review questions and/or objectives.	6
*Methods*			
Protocol and registration	5	Indicate whether a review protocol exists; state if and where it can be accessed (e.g., a Web address); and if available, provide registration information, including the registration number.	N/A
Eligibility criteria	6	Specify characteristics of the sources of evidence used as eligibility criteria (e.g., years considered, language, and publication status), and provide a rationale.	8–9
Information sources*	7	Describe all information sources in the search (e.g., databases with dates of coverage and contact with authors to identify additional sources), as well as the date the most recent search was executed.	7–10
Search	8	Present the full electronic search strategy for at least 1 database, including any limits used, such that it could be repeated.	7–9
Selection of sources of evidence†	9	State the process for selecting sources of evidence (i.e., screening and eligibility) included in the scoping review.	8–9
Data charting process‡	10	Describe the methods of charting data from the included sources of evidence (e.g., calibrated forms or forms that have been tested by the team before their use, and whether data charting was done independently or in duplicate) and any processes for obtaining and confirming data from investigators.	9
Data items	11	List and define all variables for which data were sought and any assumptions and simplifications made.	7–11
Critical appraisal of individual sources of evidence§	12	If done, provide a rationale for conducting a critical appraisal of included sources of evidence; describe the methods used and how this information was used in any data synthesis (if appropriate).	N/A
Synthesis of results	13	Describe the methods of handling and summarizing the data that were charted.	7–11
*Results*			
Selection of sources of evidence	14	Give numbers of sources of evidence screened, assessed for eligibility, and included in the review, with reasons for exclusions at each stage, ideally using a flow diagram.	8–9
Characteristics of sources of evidence	15	For each source of evidence, present characteristics for which data were charted and provide the citations.	13
Critical appraisal within sources of evidence	16	If done, present data on critical appraisal of included sources of evidence (see item 12).	N/A
Results of individual sources of evidence	17	For each included source of evidence, present the relevant data that were charted that relate to the review questions and objectives.	11
Synthesis of results	18	Summarize and/or present the charting results as they relate to the review questions and objectives.	11–16
*Discussion*			
Summary of evidence	19	Summarize the main results (including an overview of concepts, themes, and types of evidence available), link to the review questions and objectives, and consider the relevance to key groups.	17–28
Limitations	20	Discuss the limitations of the scoping review process.	29
Conclusions	21	Provide a general interpretation of the results with respect to the review questions and objectives, as well as potential implications and/or next steps.	32
*Funding*			
Funding	22	Describe sources of funding for the included sources of evidence, as well as sources of funding for the scoping review. Describe the role of the funders of the scoping review.	N/A

## Appendix 2: search strategy used in Pubmed

**Table:**

	Query	Search Details	Results
#1	girls OR women OR adolescents OR teenagers OR children OR "young women" OR "young girls" OR "school girls"	"girl s"[All Fields] OR "women"[MeSH Terms] OR "women"[All Fields] OR "girls"[All Fields] OR "womans"[All Fields] OR "women"[MeSH Terms] OR "women"[All Fields] OR "woman"[All Fields] OR "women s"[All Fields] OR "womens"[All Fields] OR "adolescences"[All Fields] OR "adolescency"[All Fields] OR "adolescent"[MeSH Terms] OR "adolescent"[All Fields] OR "adolescence"[All Fields] OR "adolescents"[All Fields] OR "adolescent s"[All Fields] OR "adolescent"[MeSH Terms] OR "adolescent"[All Fields] OR "teenage"[All Fields] OR "teenager"[All Fields] OR "teenagers"[All Fields] OR "teenaged"[All Fields] OR "teenager s"[All Fields] OR "teenages"[All Fields] OR "child"[MeSH Terms] OR "child"[All Fields] OR "children"[All Fields] OR "child s"[All Fields] OR "children s"[All Fields] OR "childrens"[All Fields] OR "childs"[All Fields] OR "young women"[All Fields] OR "young girls"[All Fields] OR "school girls"[All Fields]	5,493,635
#2	"adolescent pregnancy" OR "teenage pregnancy" OR "childhood pregnancy" OR "girl Pregnancy" OR "child pregnancy" or "unintended pregnancy"	"adolescent pregnancy"[All Fields] OR "teenage pregnancy"[All Fields] OR "childhood pregnancy"[All Fields] OR "girl Pregnancy"[All Fields] OR "child pregnancy"[All Fields] OR "unintended pregnancy"[All Fields]	7731
#3	Nigeria	"nigeria"[MeSH Terms] OR "nigeria"[All Fields] OR "nigeria s"[All Fields]	69,567
#4	#1 AND #2	("girl s"[All Fields] OR "women"[MeSH Terms] OR "women"[All Fields] OR "girls"[All Fields] OR ("womans"[All Fields] OR "women"[MeSH Terms] OR "women"[All Fields] OR "woman"[All Fields] OR "women s"[All Fields] OR "womens"[All Fields]) OR ("adolescences"[All Fields] OR "adolescency"[All Fields] OR "adolescent"[MeSH Terms] OR "adolescent"[All Fields] OR "adolescence"[All Fields] OR "adolescents"[All Fields] OR "adolescent s"[All Fields]) OR ("adolescent"[MeSH Terms] OR "adolescent"[All Fields] OR "teenage"[All Fields] OR "teenager"[All Fields] OR "teenagers"[All Fields] OR "teenaged"[All Fields] OR "teenager s"[All Fields] OR "teenages"[All Fields]) OR ("child"[MeSH Terms] OR "child"[All Fields] OR "children"[All Fields] OR "child s"[All Fields] OR "children s"[All Fields] OR "childrens"[All Fields] OR "childs"[All Fields]) OR "young women"[All Fields] OR "young girls"[All Fields] OR "school girls"[All Fields]) AND ("adolescent pregnancy"[All Fields] OR "teenage pregnancy"[All Fields] OR "childhood pregnancy"[All Fields] OR "girl Pregnancy"[All Fields] OR "child pregnancy"[All Fields] OR "unintended pregnancy"[All Fields])	7475
#5	#3 AND #4	("nigeria"[MeSH Terms] OR "nigeria"[All Fields] OR "nigeria s"[All Fields]) AND (("girl s"[All Fields] OR "women"[MeSH Terms] OR "women"[All Fields] OR "girls"[All Fields] OR ("womans"[All Fields] OR "women"[MeSH Terms] OR "women"[All Fields] OR "woman"[All Fields] OR "women s"[All Fields] OR "womens"[All Fields]) OR ("adolescences"[All Fields] OR "adolescency"[All Fields] OR "adolescent"[MeSH Terms] OR "adolescent"[All Fields] OR "adolescence"[All Fields] OR "adolescents"[All Fields] OR "adolescent s"[All Fields]) OR ("adolescent"[MeSH Terms] OR "adolescent"[All Fields] OR "teenage"[All Fields] OR "teenager"[All Fields] OR "teenagers"[All Fields] OR "teenaged"[All Fields] OR "teenager s"[All Fields] OR "teenages"[All Fields]) OR ("child"[MeSH Terms] OR "child"[All Fields] OR "children"[All Fields] OR "child s"[All Fields] OR "children s"[All Fields] OR "childrens"[All Fields] OR "childs"[All Fields]) OR "young women"[All Fields] OR "young girls"[All Fields] OR "school girls"[All Fields]) AND ("adolescent pregnancy"[All Fields] OR "teenage pregnancy"[All Fields] OR "childhood pregnancy"[All Fields] OR "girl Pregnancy"[All Fields] OR "child pregnancy"[All Fields] OR "unintended pregnancy"[All Fields]))	153

## Abbreviations

AJOL: African journals online
ASRHR: Adolescent Sexual Reproductive Health and Right
CSE: Comprehensive sexuality education
DESA: United Nations Department of Economic and Social Affairs
ECOWAS: Economic community of West African States
FP: Family planning
GPECM: Global Programme to End Child Marriage
LARC: Long-acting reversible contraception
LMICS: Low- and middle-income countries
MDG: Millennium development goals
PRISMA: Preferred Reporting Items for Systematic reviews and Meta-Analyses
SDG: Sustainable
SRH: Sexual and reproductive health
SSA: Sub-Saharan Africa
UNFPA: United Nations Population Fund
UNICEF: United Nations International Children’s Emergency Fund
WHO: World Health Organization
WOS: Web of Science
